# Molecular Basis of White Adipose Tissue Remodeling That Precedes and Coincides With Hibernation in the Syrian Hamster, a Food-Storing Hibernator

**DOI:** 10.3389/fphys.2018.01973

**Published:** 2019-01-28

**Authors:** Yuichi Chayama, Lisa Ando, Yuya Sato, Shuji Shigenobu, Daisuke Anegawa, Takayuki Fujimoto, Hiroki Taii, Yutaka Tamura, Masayuki Miura, Yoshifumi Yamaguchi

**Affiliations:** ^1^Department of Genetics, Graduate School of Pharmaceutical Sciences, The University of Tokyo, Tokyo, Japan; ^2^Functional Genomics Facility, National Institute for Basic Biology, Okazaki, Japan; ^3^Department of Pharmacology, Faculty of Pharmacy and Pharmaceutical Sciences, Fukuyama University, Fukuyama, Japan; ^4^Hibernation Metabolism, Physiology and Development Group, Institute of Low Temperature Science, Hokkaido University, Sapporo, Japan

**Keywords:** hibernation, Syrian hamsters, white adipose tissue (WAT), beige adipocytes, lipid metabolism

## Abstract

Mammalian hibernators store fat extensively in white adipose tissues (WATs) during pre-hibernation period (Pre-HIB) to prepare for hibernation. However, the molecular mechanisms underlying the pre-hibernation remodeling of WAT have not been fully elucidated. Syrian hamsters, a food-storing hibernator, can hibernate when exposed to a winter-like short day photoperiod and cold ambient temperature (SD-Cold). Animals subjected to prolonged SD-Cold had smaller white adipocytes and beige-like cells within subcutaneous inguinal WAT (iWAT). Time-course analysis of gene expression with RNA-sequencing and quantitative PCR demonstrated that the mRNA expression of not only genes involved in lipid catabolism (lipolysis and beta-oxidation) but also lipid anabolism (lipogenesis and lipid desaturation) was simultaneously up-regulated prior to hibernation onset in the animals. The enhanced capacity of both lipid catabolism and lipid anabolism during hibernation period (HIB) is striking contrast to previous observations in fat-storing hibernators that only enhance catabolism during HIB. The mRNA expression of mTORC1 and PPAR signaling molecules increased, and pharmacological activation of PPARs indeed up-regulated lipid metabolism genes in iWAT explants from Syrian hamsters. These results suggest that the Syrian hamster rewires lipid metabolisms while preparing for hibernation to effectively utilize body fat and synthesize it from food intake during HIB.

## Introduction

**FIGURE 1:**
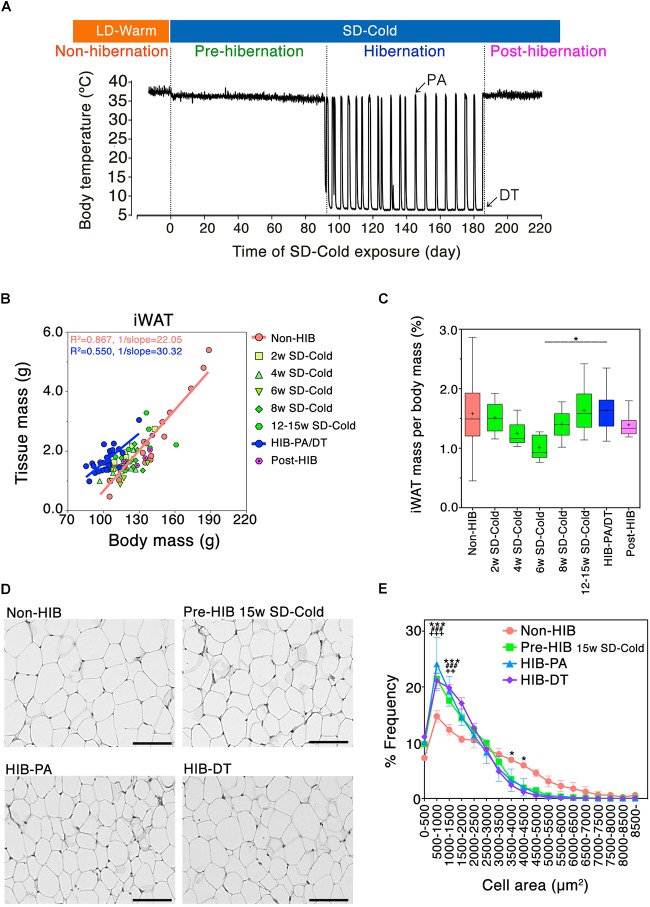
Mass and morphological changes of iWAT during the transitional period from non-hibernation state to hibernation state. **(A)** A representative change in core body temperature of hamsters measured by intraperitoneally implanted core body temperature loggers. At Post-hibernation, animals spontaneously terminated hibernation while still being kept in SD-Cold conditions. **(B)** Positive correlation of adipose tissue mass with body mass of hamsters: orange line; Non-HIB group, *R*^2^ = 0.867, 1/slope = 22.05, *P* < 0.0001: blue line; HIB-PA/DT group; *R*^2^ = 0.550, 1/slope = 30.32, *P* < 0.0001. Non-HIB, non-hibernation group under LD-Warm conditions (*n* = 20); 2/4/6/8/12–15 weeks SD-Cold, pre-hibernation group at different time points after the transfer to an SD-Cold environment (*n* = 8, 8, 5, 8, and 8, respectively); HIB-PA/DT, hibernation group combing both PA and DT (*n* = 21); Post-HIB, post-hibernation group (*n* = 7). **(C)** Changes of iWAT-to-body mass ratio over time. Horizontal lines and crosses indicate medians and means of iWAT-to-body mass ratio, respectively. Boxes enclose the interquartile ranges and whiskers show the minimum and maximum iWAT-to-body mass ratio of each experimental group. ^∗^*p* < 0.05 between the “6 weeks SD-Cold” and “HIB-PA/DT,” assessed by one-way ANOVA followed by analysis using Tukey’s multiple comparison tests. **(D,E)** H&E staining **(D)** and the frequency distribution of adipocyte cell size **(E)** in iWAT from the four different states (*n* = 3 for each condition). The results are expressed as mean ± SEM. *P*-value was derived using two-way ANOVA and Bonferroni post-tests. Significant differences are marked by ^∗^ between “HIB-DT” and “Non-HIB,” ^#^ between “HIB-PA” and “Non-HIB,” and by + between “Pre-HIB” and “Non-HIB”. ^∗,^^#,^^+^*p* < 0.05, ^∗∗,^^##,^^++^*p* < 0.01, ^∗∗∗,^^###,^^+++^*p* < 0.005.

Hibernation is an adaptive strategy that allows animals to persist in environments with seasonal or unpredictable decreases in food availability (Heldmaier et al., 2004; Geiser, 2013; Nowack et al., 2017). In small-bodied mammals (e.g., hamster, ground squirrel, and chipmunk), HIB involves multiday hypothermic deep torpor bout (HIB-DT) and normothermic periodic arousal (HIB-PA) (Figure 1A). HIB-DT is characterized by the profound suppression of metabolism, body temperature, heart rate, food intake, and locomotive activity (Lyman, 1958; Melvin and Andrews, 2009; Ruf and Geiser, 2015). The drastic alterations in physiology associated with deep torpor bout - PA cycles would lead to multiple organ dysfunction and death in non-hibernators such as mice and humans, whereas hibernators can tolerate these physiological extremes. Interestingly, however, this amazing tolerance is only operational during the hibernation season in several strictly seasonal hibernators (Kondo and Shibata, 1984; Carey et al., 2003; Kurtz et al., 2006; Andres-Mateos et al., 2013). These lines of evidence suggest that hibernators prepare for hibernation by undergoing “fall transition” or in other words, “pre-hibernation remodeling,” through which their bodies are converted from the summer phenotype to the winter phenotype during fall, the pre-HIB (Kondo, 1987; Grabek et al., 2011; Jinka et al., 2011; Hindle and Martin, 2014). However, the molecular bases underlying the induction and regulation of pre-hibernation remodeling, as well as hibernation itself, remain poorly understood (Jastroch et al., 2016).

Nutritional and environmental changes induce “WAT remodeling,” which is defined as alterations in the number, shape, type, and function of the adipose resident cells (Choe et al., 2016). For instance, chronic cold stimuli can recruit beige adipocytes to the WAT of non-hibernators such as mice, rats, and humans (Wu et al., 2012; Jimenez-Aranda et al., 2013). Beige adipocytes resemble brown adipocytes in the heat-producing organ, brown adipose tissue (BAT); they contain enriched mitochondria and multilocular lipid droplets, and express mitochondrial uncoupling protein 1 (Ucp1), a key contributor to heat generation in beige and brown adipocytes in those species. These properties enable the beige and brown adipocytes to produce heat via a non-shivering thermogenesis, and the recruitment of beige adipocyte leads to the acquisition of thermogenic properties in WAT, just as BAT (Enerback et al., 1997; Harms and Seale, 2013; Okamatsu-Ogura et al., 2013; Bartesaghi et al., 2015; Kajimura et al., 2015). Although it is not yet known to our knowledge whether beige adipocytes are recruited in hibernators, WAT is expected to play a key role during multi-day fasting and the re-warming process in hibernation (Moreau-Hamsany et al., 1988; Carneheim et al., 1989; Florant et al., 2004).

During the pre-hibernation remodeling, strictly seasonal hibernators, such as marmots, dormice, ground squirrels, and bears, fatten rapidly in order to store fat reserves for survival during HIB (Armitage et al., 1976; Swenson et al., 2007; Sheriff et al., 2013; Giroud et al., 2014; Arnold et al., 2015). The expression and activity of lipogenic enzymes in WAT is increased during the pre-HIB, but once winter hibernation begins, lipogenesis is weakened, and lipolysis is dominantly activated in WAT in these strictly seasonal hibernators (Mostafa et al., 1993; Wang et al., 1997; Hampton et al., 2011; Arnold et al., 2015; Shimozuru et al., 2016). This is possibly because most of these animals studied are fat-storing hibernators; they become hyperphagia from late summer to fall, and then completely stop feeding and largely subsist on the accumulated fat as substrate for energy metabolism in winter, where respiratory quotient values are ∼0.7 (Wilson et al., 1992; Melvin and Andrews, 2009; Toien et al., 2011). In contrast to the aforementioned fat-storing hibernators, Syrian (golden) hamsters (*Mesocricetus auratus*) are food-storing hibernators that eat food stored in the nest during PA. It was reported that they maintain a high lipogenic capacity of WAT in both Pre-HIB and HIB in response to prolonged cold (Baumber and Denyes, 1963). Despite this difference between fat-storing and food-storing hibernators, the above studies suggest that dynamic reorganization of lipid metabolic pathways is a key feature of pre-hibernation remodeling in mammalian hibernators examined so far, whereas it is yet unclear how the reorganization is achieved.

To study pre-hibernation remodeling and hibernation in the laboratory, Syrian hamster is a good experimental animal model for the following reasons. Syrian hamsters can initiate hibernation throughout the year if they are maintained under appropriate winter-like conditions, and are therefore classified as opportunistic (facultative) hibernators (Jansky et al., 1984; Tamura et al., 2005; Lewis et al., 2012; Geiser, 2013; Chayama et al., 2016; Trefna et al., 2017). In the laboratory, after transfer from summer-like conditions [LD photoperiod and warm ambient temperature (LD-Warm)] to winter-like conditions [SD photoperiod and cold ambient temperature (SD-Cold)], most Syrian hamsters require a long pre-HIB (typically 2–3 months) before initiating hibernation (Figure 1A) (Jansky et al., 1984; Chayama et al., 2016). This requirement of a long induction period in SD-Cold implies that the pre-hibernation remodeling process, involving changes in the thermoregulatory system and the regulation of body mass, is present in Syrian hamsters, as in strictly seasonal hibernators (Arnold et al., 2011; Olson et al., 2013; Sheriff et al., 2013; Drew et al., 2017). In fact, during the pre-HIB, the female Syrian hamsters underwent physiological body remodeling; they gradually reduce their body mass and the set-point of core body temperature (Arai et al., 2005; Chayama et al., 2016). After the body mass dips lower than the threshold for hibernation, the hamsters begin to hibernate (Supplementary Figure S1A) (Chayama et al., 2016). However, little is known about molecular and cellular aspects of the pre-hibernation remodeling; differences between summer-like non-hibernating body conditions and winter-like hibernating body conditions, and mechanisms underlying the regulation of those pre-hibernation remodeling in Syrian hamsters.

In the present study, we hypothesized that pre-hibernation remodeling would be induced in WAT, the central organ in regulation of the whole body-energy homeostasis and body mass, under prolonged SD-Cold condition before initiating hibernation. To examine this at the cellular and molecular levels, we conducted morphological analyses of adipocytes and comprehensive RNA-seq profiling on the iWAT of female Syrian hamsters. In particular, we focused on (i) alterations in adipocyte size, and emergence of distinct types of adipocytes in iWAT, and (ii) lipid metabolism remodeling in iWAT from pre-HIB to HIB.

## Materials and Methods

### Ethics Statement

All animal work has been conducted according to the ethics guidelines of the University of Tokyo and Hokkaido University, and was approved by the Ethics Committee of the University of Tokyo (Ethical Approval no. P24–34) and of the Hokkaido University (Ethical Approval no. 18-0140).

### Animals and Housing

Female Syrian hamsters (*Mesocricetus auratus*) from a closed breeding colony were purchased from SLC, Inc., Japan. Animals were housed in groups of 3 or 4 per cage with *ad libitum* access to diets (MR standard diet, Nihon Nosan, Japan) and water under LD-Warm conditions (light condition = 16L:8D cycle, lights on 05:00–21:00, ambient temperature = 24–25°C). For hibernation induction, animals were reared under LD-Warm conditions until most animals weighed over 100–120 g. The body mass of animals and amounts of food consumption were measured weekly when cages were changed.

One to 2 weeks before the transfer to SD-Cold conditions, core body temperature (*Tb*) loggers (iButton^®^, Maxim Integrated, United States, #DS1992 L-F5 model; operating temperature range from -40 to +70°C) coated with rubber (Plasti Dip, Performix^®^; total mass ∼3.5 g, 1.8–3.5% of animal mass) were implanted intraperitoneally under inhalation anesthesia with 4% isoflurane (DS Pharma Animal Health, Japan) and intraperitoneal injection of pentobarbital sodium (65 mg/kg, diluted with phosphate-buffered saline; Kyoritsu Seiyaku, Japan). We did not calibrate iButtons as we assumed linearity and no drift of the temperature signal based on our previous experience. After 1–2 weeks of recovery, animals were transferred to SD-Cold conditions (8L:16D cycle, lights on 10:00–18:00, ambient temperature = 5°C). Animals were individually housed in polypropylene cages, and the *Tb* of animals were measured every 90 min with an accuracy of 0.5°C. The onset of hibernation was detected comprehensively by the characteristic postures of animals (rolled into ball, Supplementary Figure S1A), the reduced activity and consumption of food when cages were changed. The ‘saw-dust method’ was also used for confirming that animals successfully hibernated; wood chips placed on the back of hibernating individuals remained in place until the animals experienced HIB-PA (Jansky et al., 1984). The cage replacement and body mass measurement of animals in HIB-DT were skipped to avoid disturbing HIB-DT. HIB-DT is defined as prolonged hypothermia, with *Tb* lowered to 15°C or less (Ruf and Geiser, 2015).

*Tb* loggers were recovered from animals sacrificed by decapitation after they were subjected to 10–15 min anesthesia with intraperitoneal injection of pentobarbital sodium (65 mg/kg) and inhalation of 4% isoflurane.

### iWAT Collection

To examine the time-course of remodeling in iWAT during the pre-HIB, iWAT of hamsters was collected at different time-points: Non-HIB (euthermic and active animals at the LD-Warm condition), Pre-HIB (2, 4, 6, 8, and 12–15 weeks of the SD-Cold), HIB-PA [animals that spontaneously aroused from HIB-DT to euthermic state (*Tb* > 35°C) for 10–15 h under SD-Cold], HIB-DT (*Tb* < 15°C over 6 h), and post-hibernation group (Post-HIB: euthermic and active after spontaneous termination of hibernation under the SD-Cold). All animals were sacrificed between 13:00 and 15:00, by decapitation 10–15 min under anesthesia with intraperitoneal injection of pentobarbital sodium (65 mg/kg) and 4% isoflurane. Whole iWAT pads were dissected from the surrounding tissues, and cleaned of any contaminating tissue (Supplementary Figure S1B). The wet tissue mass of both sides of iWAT was measured for each hamster, and the average of these measurements was calculated for analysis. The correlation of iWAT mass with body mass was analyzed using Pearson’s correlation coefficient. The iWAT-to-body mass ratio for each hamster was calculated and expressed as a percentage of body mass. *P*-value was derived using one-way ANOVA and Tukey’s multiple comparison tests using the Graph Pad Prism 5.0 software (GraphPad, San Diego, CA, United States).

### Measurement of Adipocyte Size

iWAT were obtained from animals sacrificed at four collection points: Non-HIB (18 weeks old), Pre-HIB at 15 weeks of the SD-Cold condition (24 weeks old), HIB-PA (25–26 weeks old), and HIB-DT (24–26 weeks old). Three animals for each group were analyzed. One side of the iWAT was dissected into four parts (Supplementary Figure S1B), and fixed in 4% of phosphate buffered paraformaldehyde (PFA) for more than 2 days at 4°C, dehydrated, and embedded in paraffin as previously described (Berry et al., 2014). Regions 2 and 3 of iWAT were then cut in 6-μm sections and stained using hematoxylin (Sigma-Aldrich) and eosin (Wako).

Tile-scanned images of each section were obtained at 20× magnification using an AF6000 microscope (Leica Microsystems). Adipocyte cell size was semi-automatically determined using the FIJI plug-in Adiposoft (Galarraga et al., 2012). Measurements were obtained from three animals per group; two sections for each hamster were analyzed, and the mean of these measurements was used for the calculation. The frequency distribution of adipocyte sizes in iWAT was calculated and expressed as a percentage of total adipocytes counted. The results are expressed as mean ± SEM. *P*-value was derived from two-way ANOVA and Bonferroni’s multiple comparison tests using the Graph Pad Prism 5.0 software.

### Immunohistochemical Analysis

Six-micrometer sections from paraffin-embedded iWAT were deparaffinized and rehydrated, and submitted to heat antigen retrieval using a microwave oven and citrate buffer, pH 6.0 for 15 min. The endogenous peroxidase activity was quenched by exposing sections to 3% hydrogen peroxide in methanol overnight at 4°C. After rinsing extensively using Tris-buffered saline with 0.1% Tween20 (WAKO) (TBS-T), the sections were blocked using 20% ImmunoBlock (DS Pharma, Osaka, Japan) in TBS-T for 30 min at room temperature, and incubated with rabbit anti-human UCP1 polyclonal antibody (Abcam, ab23841, lot#GR116982-1) at 1:100 dilution in Immunoreaction Enhancer Solution (Can Get Signal A solution, TOYOBO) with 10% ImmunoBlock at 4°C overnight. Mouse anti-rabbit immunoglobulin G conjugated with horseradish peroxidase (Abcam, ab99702) was applied at 1:200 dilution in the Immunoreaction Enhancer Solution with 10% ImmunoBlock for 60 min at room temperature. After rinsing with Tris-buffered saline (TBS), slides were incubated with diaminobenzidine (Sigma Aldrich) and hydrogen peroxide in TBS (pH 7.2) for 15 min, and then rinsed in tap water. After counterstaining with hematoxylin (Sigma-Aldrich), the sections were dehydrated, coverslipped, and imaged at 20×/40× magnification with an AF6000 microscope (Leica Microsystems). The numbers of animals for immunohistochemical analysis were as follows; Non-HIB (*n* = 6), Pre-HIB (*n* = 6), HIB-PA (*n* = 3), and HIB-DT (*n* = 3).

### RNA Isolation and Real-Time Quantitative PCR

A whole iWAT pad dissected as described above was minced and incubated in RNA stabilization solution (RNA later, Ambion), and stored at -80°C until RNA purification. Total RNA was purified from a whole iWAT using Qiazol Lysis Reagent and RNeasy Mini Kit (Qiagen).

Total RNA samples from individual animals were quantified with a Nanodrop (Thermo Scientific). Total RNA (100–250 ng) was reverse-transcribed using a Prime Script RT reagent with a gDNA eraser (TAKARA). Primers were designed using an online tool, Primer-BLAST^1^ based on known Syrian hamster sequences (*Mesocricetus auratus*, taxid:10036). Primer sequences are depicted in Supplementary Table S6.

Quantitative real-time PCR (qPCR) was performed using a SYBR Premix Ex Taq II (Tli RnaseH Plus) (TAKARA) and a Light Cycler 480 real-time PCR system (Roche), according to the manufacturer’s instructions. The specificity of PCR product was checked using melting curve analysis. The relative mRNA expression of each gene was quantified using a standard curve. The most stable reference gene was determined from potential reference genes by geNorm analysis (Otis et al., 2010), resulting in the selection of Peptidylprolyl isomerase A (*Ppia*) as an internal control gene for normalization. Results of relative quantification are expressed as mean ± standard errors (SEM), or box-and-whisker plot. *P*-value was derived from one-way ANOVA and Tukey’s multiple comparison tests using Graph Pad Prism 5.0 software.

### RNA Isolation for Illumina Sequencing Analysis

For RNA-sequencing analysis, three animals were sacrificed per collection point (Non-HIB, Pre-HIB, HIB-PA, and HIB-DT, 12 animals) (Figure 3A) and processed for tissue collection. Total RNA was purified from whole iWAT as described above. Total RNA samples from individual animals were quantified on a Nanodrop (Thermo Scientific) and Qubit RNA BR assay kit (Invitrogen). Genomic DNA remaining in purified RNA samples was measured using a Qubit dsDNA HS assay kit (Invitrogen), and confirmed to be lower than 10% of total RNA. Quality of total RNA was evaluated using capillary electrophoresis (Agilent Bioanalyzer 2100). All the 12 samples were verified as high quality (RNA Integrity Number > 7.9) and thus were converted to Illumina sequencing libraries.

### Library Creation

Conversion of RNA samples to sequencing libraries was performed using TruSeq Sample Preparation Kit v2 (Illumina; RS-122-2001) according to manufacturer’s protocol, with minor modifications: all reactions were carried out at half scale, fragmentation of mRNA was performed for 4 min, and PCR reduced to 8 cycles to minimize PCR biases. One microgram of total RNA was used for each library. The size distribution of the libraries was validated using capillary electrophoresis (Agilent Bioanalyzer 2100) and quantified using KAPA library quantification kits (KAPA Biosystems) using a Light Cycler 480 real-time PCR system (Roche).

### Sequencing

Twelve multiplexed libraries were sequenced in one lane using HiSeq1500 (Illumina) with 101-bp paired-end readings. Raw data processing, base calling, and quality control were performed according to manufacturer’s standard protocol using RTA, OLB, and CASAVA software (Illumina). All raw Illumina sequences are available for download at the DDBJ Sequence Read Archive (DRA) under the accession number PRJDB6278.

### Bioinformatic Analysis

Sequencing using Illumina HiSeq1500 platform yielded 141.3 million 101-bp paired-end sequence reads (Supplementary Table S1). Quality of the raw sequence data was validated by FastQC. Sequence reads were mapped to a Syrian hamster genomic scaffold and annotation^2^ using Tophat v2.0.10 (Supplementary Table S2). To quantify gene expression and determine genes that are differentially expressed across the four groups, the *q*-values (adjusted *P*-value) of test statistics for each gene were computed using Cufflinks v2.2.1. The Cufflinks outputs were further analyzed and visualized using cummeRbund and Morpheus^3^ (Broad Institute). Hierarchical clustering was performed by one minus pearson correlation.

### Adipose Tissue Explants Culture

iWATs were dissected out from hamsters of Non-HIB group (euthermic and active at the LD-Warm condition, 28–31 weeks old, *n* = 5) and HIB-DT group (heterothermic and inactive at the SD-Cold condition, 28–31 weeks old, *n* = 5). Adipose explant cultures were established by modification of a reported procedure (Toyoda et al., 2008). The tissues were minced into small fragments in cold PBS, then were transferred to a tube containing 30 mL of Dulbecco’s Modified Eagle’s Medium (DMEM; D5921, Sigma-Aldrich) with 8 mM glutamine (Sigma-Aldrich), 1% (v/v) penicillin/streptomycin (pe/st) solution (WAKO), and 1% (w/v) bovine serum albumin (BSA) (A7030; Sigma-Aldrich), and were gently pre-cultured with rotation for 6 h in a CO_2_ incubator (37°C, 5% CO_2_). The tissue fragments were placed into 24-well plates containing 800 μL of DMEM (8 mM glutamine, 1% pe/st, 1% BSA), and treated with or without 0.1% dimethyl sulfoxide (DMSO) (WAKO), 100 μM fenofibrate (Wako), 100 μM L-165041 (Cayman), and 100 μM rosiglitazone (Tokyo Kasei), for 48 h in the CO_2_ incubator. The culture medium was daily replaced with fresh agonist-containing medium. Four technical replicates were used for each treatment condition. After the treatment with PPAR agonists, tissue fragments were collected in RNA stabilizing solution, and used for RNA analysis as described above.

Mean of fold change of the four technical replicates was calculated using Microsoft Excel. Standardization among five biological replicates was performed as previously described (Willems et al., 2008). Boxes enclose the interquartile ranges and whiskers show the minimum and maximum values of log2-fold change of each experimental group against non-treated group. *P*-value was derived from one-way ANOVA and Tukey’s multiple comparison tests using Graph Pad Prism 5.0 software (GraphPad).

## Results

### Utilization and Preservation of White Adipose Tissues During the Pre-hibernation and Hibernation Periods

To examine how WAT is remodeled during the pre-HIB in Syrian hamsters, we focused on iWAT (Supplementary Figure S1B). Previously, we demonstrated that prolonged SD-Cold condition reduces the body mass of female Syrian hamsters raised in LD-Warm condition below the threshold for initiating hibernation under *ad lib* feeding (Chayama et al., 2016). We found that iWAT mass strongly correlated with the body mass of hamsters under LD-Warm conditions (Figure 1B, Non-HIB group). Under SD-Cold conditions, iWAT-to-body mass ratio tended to decrease, and minimized 6 weeks after the transition (Figure 1C). Interestingly, however, this ratio did not continue to fall during the SD-Cold period; rather, an increase was observed in animals exposed to SD-Cold for 8–15 weeks and which had entered hibernation (Figure 1C). This trend was also observed in intraperitoneal WATs (Supplementary Figure S1C). These data indicate that iWAT mass was lost extensively at first under SD-Cold conditions, but was then retained relative to overall body mass loss.

We then examined the histological changes of iWAT during hibernation. The proportion of small adipocytes (area: 500–1500 μm^2^) was significantly greater in hibernating animals (HIB-PA and HIB-DT groups) than in the Non-HIB group (Figures 1D,E). This increased number of small white adipocytes was already evident in the Pre-HIB group (Figures 1D,E). Because adipocyte size can reflect changes in the mobilization or accumulation of stored lipids, these data suggest that Syrian hamsters alter lipid metabolism in iWAT under prolonged SD-Cold conditions prior to hibernation.

### UCP1-Positive, Beige-Like Multilocular Adipocytes Emerge in iWAT During the Pre-hibernation Period

**FIGURE 2:**
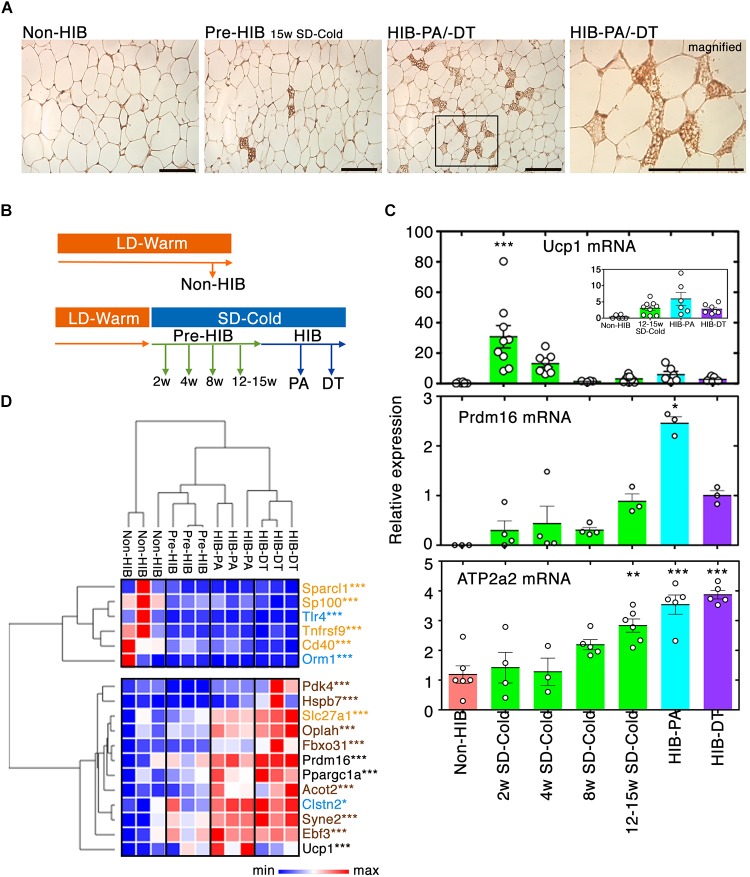
Recruitment of beige-like cells to iWAT by prolonged SD-Cold exposure. **(A)** Representative images of UCP1 staining of iWAT from each condition. The numbers of iWAT with beige-like cells in Non-HIB, Pre-HIB, HIB-PA, and HIB-DT were 0/6, 5/6, 2/3, and 2/3, respectively. Scale bars, 100 μm. **(B,C)** Time-course analysis of *Ucp1* and *Prdm16* mRNA expression in iWAT by qPCR. **(B)** Schematic overview of time-course analysis. **(C)** Relative expression of *Ucp1*, *Prdm16*, and *Atp2a2* mRNA expression. The numbers of samples analyzed for *Ucp1* mRNA were Non-HIB: *n* = 6, 2/4/8/12–15 weeks SD-Cold: *n* = 9, 8, 4, and 8, respectively, HIB-PA: *n* = 6, and HIB-DT: *n* = 7. Those for *Prdm16* mRNA were Non-HIB: *n* = 3, 2/4/8/12–15 weeks SD-Cold: *n* = 4, 4, 4, and 3, respectively, HIB-PA: *n* = 3, and HIB-DT: *n* = 3. Those for *Atp2a2* mRNA were Non-HIB: *n* = 6, 2/4/8/12–15 weeks SD-Cold: *n* = 4, 3, 5, and 6, respectively, HIB-PA: *n* = 5, and HIB-DT: *n* = 5. The results are expressed as mean ± SEM. White circles show each animal. *P*-value was derived using one-way ANOVA and Tukey’s multiple comparison tests. ^∗^*p* < 0.05, ^∗∗^*p* < 0.01, ^∗∗∗^*p* < 0.005 against the “Non-HIB group.” **(D)** Heat map representation of relative expression changes of genes in the beige/brown adipocyte signature as differentially expressed with *q*-value ≤ 0.01 and more than twofold change between any groups. Blue, orange, and brown characters indicate marker genes for WAs, beige adipocytes, and brown adipocytes, respectively. Pre-HIB here indicates pre-hibernation group following 15 weeks of exposure to SD-Cold conditions. ^∗^*q* < 0.05, ^∗∗^*q* < 0.01, ^∗∗∗^*q* < 0.005.

Prolonged cold exposure induces beige adipocyte formation in WAT of non-hibernators including mice, rats, and humans. However, few studies have addressed whether hibernators recruit beige adipocytes to their WAT. Our immunohistochemical analysis on WAT for UCP1 identified a small proportion of multilocular beige-like cells positive for UCP1 in iWAT of pre-hibernation and hibernating hamsters under prolonged SD-Cold conditions (Figure 2A). Time-course analysis using qPCR revealed that expression of *Ucp1* mRNA increased drastically 2 weeks after initiation of SD-Cold conditions (Figures 2B,C). It was then gradually reduced by the onset of hibernation (Figure 2C), although it still remained higher than levels found under LD-Warm conditions (Non-HIB).

Besides, the mRNA expression of PR-domain containing 16 (*Prdm16*), a master regulator of beige adipocyte differentiation in WAT (Cohen et al., 2014), was induced 15 weeks after SD-Cold exposure, and significantly up-regulated in hibernating animals (Figure 2C). Moreover, *Atp2a2* gene (encoding SERCA2) was significantly up-regulated in iWAT after SD-Cold exposure (Figure 2C). Recently, SERCA2 was shown to be up-regulated in iWAT from transgenic mice overexpressing *Prdm16* in an adipose tissue-selective manner (Ikeda et al., 2017). Importantly, it was further shown to be crucial for a non-canonical UCP1-independent thermogenic mechanism through Ca^2+^ cycling in beige adipocyte of mice, pigs, and humans (Ikeda et al., 2017). Thus, the beige-like cells might generate heat during hibernation by activating the enhanced SERCA2 pathway rather than UCP1 pathway in iWAT.

Further evidence that beige-like cells emerged in iWAT after SD-Cold exposure, was provided by a comprehensive gene expression analysis following RNA-seq (see below); a ‘beige/brown adipocyte’ signature (Wu et al., 2012; Harms and Seale, 2013; Long et al., 2014; de Jong et al., 2015; Shinoda et al., 2015) was indeed induced in hibernating animals (Figure 2D). Altogether, these data indicate that beige-like cells are recruited to the iWAT of Syrian hamsters after prolonged exposure to SD-Cold, and during the HIB.

### Global Gene Expression Profiling of White Adipose Tissue Discriminates Hibernation State From Non-hibernation State

**FIGURE 3:**
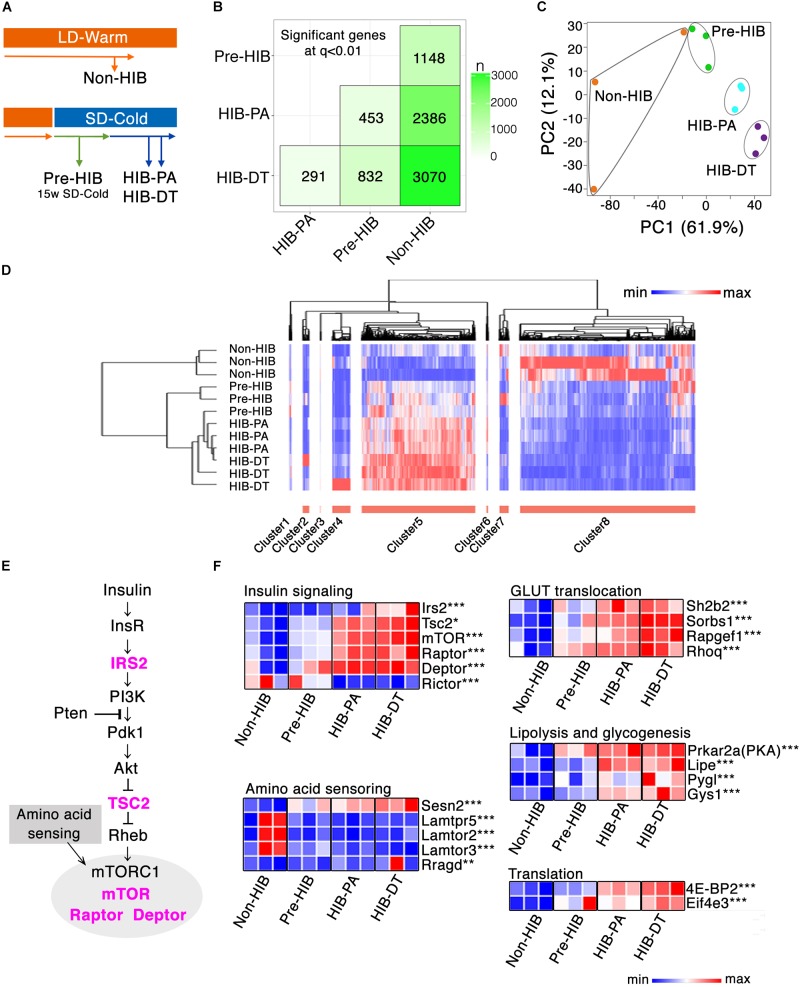
Global gene expression profiling of iWAT. **(A)** Schematic overview of the four experimental groups that were used for RNA-seq analysis of iWAT (*n* = 3 per group). **(B)** SigMatrix plot indicating the number of DEGs among each combination of groups at 0.01 *q*-value threshold. **(C)** Principle component analysis on 3804 genes. PC1, principle component 1; PC2, principle component 2. **(D)** Hierarchal clustering analysis on the screened 2831 DEGs, which exhibited more than twofold change between any groups with *q*-value ≤ 0.01. Relative mRNA expression level among four groups are denoted in red (high expression) to blue (low expression) gradient. **(E,F)** mRNA expression changes of genes involved in insulin signaling. **(E)** Insulin-Akt-mTORC1 signaling cascade. The genes colored in magenta denote the significant up-regulation either in HIB-PA or HIB-DT compared to the Non-HIB group. **(F)** Heat map representation of relative mRNA expression change in the Insulin-Akt-mTORC1 signaling pathway and its downstream signaling pathways. ^∗^*q* < 0.05, ^∗∗^*q* < 0.01, ^∗∗∗^*q* < 0.005.

To further identify differences in iWAT between the non-hibernation and hibernation states, we conducted RNA-seq analysis (Figure 3A). More than 8 million high-quality reads of transcripts were obtained in all the groups, and ≥70% of them were successfully mapped on hamster’s genome and quantified (Supplementary Tables S1, S2). Pairwise comparison of transcripts profile showed the positive correlations of most gene expressions between each of the groups and subsets of the genes differentially expressed (Supplementary Figures S2, S3). Total 3804 genes were identified as differentially expressed genes (DEGs) between each of the groups, with a *q*-value of 0.01. The number of DEGs between two groups is shown in Figure 3B and Supplementary Figure S3.

To visualize the variation and patterns of gene expression, principal component analysis (PCA) and hierarchal clustering analysis of the DEGs were performed. Principal component 1 (PC1) clearly described the transition from non-hibernation to hibernation states (Figure 3C). Among the genes that contributed the most to PC1, those involved in insulin signaling (e.g., *Rapgef1*, *Calm2*, *Exoc7*, *mTOR*, *Prkar2a*, *Rhoq*, *Rps6*; Gene name for each gene symbol described hereafter is provided in Supplementary Table S7) were identified as significantly enriched upon Kyoto Encyclopedia of Genes and Genomes (KEGG) pathway analysis (Supplementary Table S3). Hierarchical clustering analysis of 2831 DEGs, which exhibited more than twofold changes of expression between at least any two groups, also identified distinct transcript profiles between groups (Figure 3D). The Pre-HIB group was closer to the cluster of hibernation groups (HIB-PA and HIB-DT groups) than was the Non-HIB group (Figure 3D). These data demonstrate that the iWAT gene expression profile in Syrian hamsters is altered during pre-hibernation remodeling. To gain further insight into pre-hibernation remodeling, we focused on clusters 5 and 8, which discriminate the non-hibernation state from the others under prolonged SD-Cold conditions (Pre-HIB, HIB-PA, and HIB-DT; Figure 3D).

Cluster 8, which was down-regulated in hibernation groups, consisted of 1528 genes including a number of ribosomal protein genes and genes related to immune cell signaling (e.g., primary immunodeficiency, chemokine signaling, cytokine–cytokine receptor interaction; Supplementary Table S4). The down-regulation of immune-cell signature genes might be indicative of changes in the abundance of resident cells (e.g., pre-adipocytes, macrophages, T-cells, and B-cells) in iWAT during the transition period (Osborn and Olefsky, 2012; Choe et al., 2016).

The 990 genes of cluster 5, which was up-regulated in hibernation groups, contained many genes involved in cell–cell interaction (e.g., focal adhesion, ECM-receptor interaction, adherens junctions, and vascular smooth muscle contraction), which may reflect progression of angiogenesis in iWAT during the pre-HIB (Supplementary Table S5). Other enriched pathways in cluster 8 include insulin signaling, peroxisome proliferator-activated receptor (PPAR) signaling, and adipocytokine signaling pathways. These pathways are known to regulate the transcription of genes related to energy metabolism, adipogenesis, mitochondrial biogenesis, and ribosome biogenesis (Cai et al., 2016). Together, these results show that the expression profiles of genes involved in several pathways are concomitantly regulated with the progression of pre-hibernation remodeling, and these profiles can be used as a predictive signature to discriminate between non-hibernation and hibernation states.

Among the genes involved in the insulin signaling that were enriched in cluster 8, mechanistic target of rapamycin complex 1 (mTORC1) (*mTOR, Rptor*, and *Dptor*), which responds to nutrient signals through amino acid sensing pathway and insulin-PI3K-Akt signaling pathway, were significantly up-regulated in hibernation groups (HIB-PA and HIB-DT) than in the non-hibernation group (non-HIB) (Figures 3E,F). Among the key molecular players in the amino acid sensing pathway (Ricoult and Manning, 2013; Lee et al., 2017), Sestrin2 (*Sesn2*) was significantly up-regulated in the hibernation groups (Figure 3F). Furthermore, the downstream targets of PI3K-Akt and mTORC1 signaling networks were also up-regulated by prolonged exposure to SD-Cold conditions. Genes involved in GLUT translocation (*Sh2b2, Sorbs1, Rapgef1*, and *Rhoq*), lipolysis and glycogenesis (*Prkar2a, Lipe, Pygl*, and *Gys1*), and translation (*Eif4e3* and *4E-BP2*) were mostly up-regulated in hibernation groups (Figure 3F). In sum, these changes in gene expression appear to enhance the functional capacity of the mTORC1 pathway and its downstream signaling networks.

### Lipid Catabolism and Anabolism Are Both Increased in iWAT Prior to Hibernation

**FIGURE 4:**
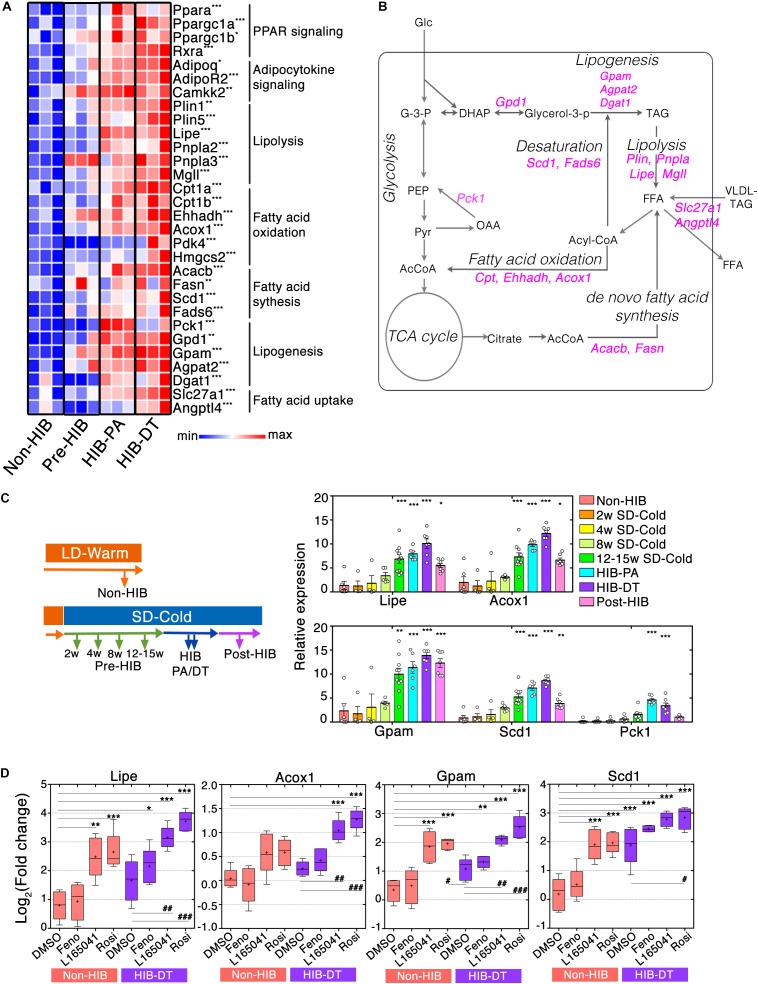
PPAR signaling augments mRNA expression of genes required for lipid catabolism and anabolism in iWAT during hibernation. **(A)** Heat maps of fold changes in mRNA expression of genes related to PPAR signaling pathways. ^∗^*q* < 0.05, ^∗∗^*q* < 0.01, ^∗∗∗^*q* < 0.005. **(B)** A schematic representation of the cellular biochemical networks of lipid and glucose metabolism in WAT. Genes that encode enzymes for chemical reaction and that are up-regulated during HIB are shown in magenta. AcCoA (acetyl-CoA), DHAP (dihydroxyacetone phosphate), FFA (free fatty acid), G-3-P (glyceraldehyde 3-phosphate), Glc (glucose), Glycerol-3-P (glycerol 3-phosphate), OAA (oxaloacetate), PEP (phosphoenolpyruvate), Pyr (pyruvate), TAG (triacylglyceride), VLDL-TAG (very-low-density lipoprotein triacylglyceride). **(C)** Time-course analysis of the lipid metabolism genes that were up-regulated during HIB. qPCR analysis of relative mRNA expression levels of lipid metabolism genes at the different time points. The results are expressed as mean ± SEM. White circles show each animal. *P*-value was derived from one-way ANOVA and Tukey’s multiple comparison tests. ^∗^*p* < 0.05, ^∗∗^*p* < 0.01, ^∗∗∗^*p* < 0.005 against the “Non-HIB group.” **(D)** *Ex vivo* experiments of adipose explant cultures. iWAT explants from Syrian hamsters were treated with 0.1% dimethyl sulfoxide (DMSO), 100 μM fenofibrate (Feno), 100 μM L165041 (L165041), and 100 μM rosiglitazone (Rosi) for 48 h. Relative mRNA expression level of the lipid metabolism genes in the different conditions was determined using qPCR. Horizontal lines and crosses indicate medians and means of relative mRNA expression, respectively. Boxes enclose the interquartile ranges and whiskers show the minimum and maximum relative mRNA expression of each experimental condition. *P*-value was derived using one-way ANOVA and Tukey’s multiple comparison tests. ^∗^*p* < 0.05, ^∗∗^*p* < 0.01, ^∗∗∗^*p* < 0.005 against the DMSO-treated condition of the Non-HIB group. ^#^*p* < 0.05, ^##^*p* < 0.01, ^###^*p* < 0.005 against the DMSO-treated condition of HIB-DT group.

Activation of mTORC1 mutually affects the expression and activity of PPAR molecules in WAT (Laplante and Sabatini, 2009). PPARs are important nutrient sensors that regulate transcriptions of genes involved in lipid metabolism (Nelson et al., 2009; Ahmadian et al., 2013; Kersten, 2014). Genes involved in transcriptional co-regulation of PPARs (*Ppara, Ppargc1a/b*, and *Rxra/g*) and adipocytokine signaling (*Adipoq, Adipor2*, and *Camkk2*) were significantly higher in hibernation groups than in the non-hibernation group (Figure 4A).

To further address how lipid metabolism pathways are altered during pre-hibernation remodeling, we analyzed the mRNA expression of catabolic genes involved in lipolysis (*Plin1/5, Lipe, Pnpla2/3*, and *Mgll*) and fatty acid (FA) oxidation (FAO) (*Cpt1a/b, Acadvl, Ehhadh*, and *Acox1*) by RNA-seq data and qPCR. This analysis showed that they were significantly elevated in hibernation groups (Figures 4A,B and Supplementary Figure S4). Interestingly, anabolic genes involved in lipogenesis (*Gpd1, Gpam, Agpat2*, and *Dgat1*), FA synthesis (*Acacb* and *Fasn*), and desaturation (*Scd1* and *Fads6*) were also highly expressed in those groups (Figure 3A and Supplementary Figure S4). In addition, the mRNA expression of genes involved in FA uptake (*Slc27a1* and *Angptl4*) was also higher in hibernating animals (Figure 4A). These results suggest that the capacities of both lipid catabolism and anabolism are simultaneously enhanced during hibernation, which contributes to an efficient regulation of the utilization and storage of lipid fuels in iWAT (Figure 4B).

To further identify the time at which remodeling of lipid metabolic pathways initiates during pre-hibernation remodeling, we performed a time course qPCR analysis of lipid metabolism genes (Figure 4C). Four genes that are crucial for each lipid metabolic process (*Lipe* for lipolysis, *Acox1* for FAO, *Gpam* for lipogenesis, and *Scd1* for FA desaturation) were selected as readout of those pathways. This analysis revealed that no changes in the mRNA expression of all four genes were observed after 8 weeks’ exposure to SD-CA conditions (Pre-HIB of 2, 4, and 8 weeks after initiation of SD-Cold conditions), but they were significantly up-regulated after 12–15-week exposure to SD-CA conditions (Pre-HIB 12–15 weeks SD-Cold). The mRNA expression of these genes peaked during hibernation (HIB-PA and HIB-DT) (Figure 4C). Thus, up-regulation of lipid metabolism genes preceded the onset of hibernation. Interestingly, after an average of 4–6 months of hibernation, the mRNA expression of *Lipe*, *Acox1*, and *Scd1* fell to the same level as that observed in the Pre-HIB group by 12–15 weeks after initiation of SD-Cold conditions, whereas *Gpam* mRNA expression remained at higher level (Figure 4C, Post-HIB). At this timepoint, all the animals examined spontaneously terminated hibernation and started to gain body mass, even though they were still maintained under SD-Cold conditions (Figure 1A, “Post-hibernation”). These results suggest that the elevated mRNA expression of genes involved in lipolysis, FAO, and FA desaturation is a phenomenon closely associated with the winter-like body state that allows hibernation, and is not just a consequence of simple cold acclimation.

The gene encoding phosphoenolpyruvate carboxykinase 1 (*Pck1*), a key enzyme that promotes glyceroneogenesis in WAT (Beale et al., 2004), was present in cluster 5 (Figure 3D), and exhibited a “hibernation-specific” expression pattern, in that it significantly increased only in hibernation groups, but not in pre-hibernation groups (Figure 4C). After the HIB, the *Pck1* mRNA returned to the same level as that observed in the non-hibernation and pre-hibernation groups. Such hibernation-specific induction of *Pck1* may enhance the rate of glyceroneogenesis and FA recycling in order to maintain triglyceride homeostasis during hibernation (Figure 4C).

### Involvement of PPAR Signaling in Up-Regulation of Both Lipid Catabolism and Anabolisms Genes

We then explored potential mechanisms that elicit an enhanced mRNA expression of lipid metabolism genes. Isotypes of the PPAR family (PPAR-α, -β/δ, and -γ) have different expression patterns, ligand specificities, and co-regulator interactions (Gross et al., 2017). Because PPAR signaling molecules were up-regulated during the pre-HIB, we addressed whether they might be responsible for induction of the lipid metabolism genes that were highly expressed in iWAT during hibernation. PPAR agonists were applied to cultured iWAT explants obtained from non-hibernating hamsters (Non-HIB at LD-Warm) and hibernating hamsters (HIB-DT at SD-Cold). The explants from Non-HIB animals exhibited clear responses to PPAR-β/δ or -γ agonists (L-165041 or rosiglitazone, respectively), resulting in significant up-regulation of *Lipe*, *Gpam*, and *Scd1* mRNA and, to a lesser extent, *Acox1*, when compared to control (DMSO) (Figure 4D). In contrast, the PPAR-α agonist, fenofibrate did not affect the expression of any of these genes (Figure 4D). Likewise, the iWAT explants from hibernating animals (HIB-DT) exhibited significant increases of *Lipe*, *Acox1*, and *Gpam* mRNAs in response to PPAR-β/δ or -γ agonists, but the PPAR-α agonist. The basal mRNA expression of these genes was similar in DMSO-treated HID-DT and Non-HIB explants, suggesting that *ex vivo* manipulation of iWAT may mask elevated PPAR-dependent signaling in tissues from hibernating animals as shown in Figure 4. However, the basal expression of *Scd1* mRNA was significantly higher in HIB-DT explants when compared to their Non-HIB counterparts. We infer that the mechanisms that underlie up-regulation of *Scd1* mRNA during hibernation are resistant to experimental procedures, and/or that *Scd1* mRNA expression may be regulated differently than the mRNA expression of *Lipe*, *Acox1*, and *Gpam.* Consistent with this, *Scd1* mRNA in HIB-DT was further increased only by the PPAR-γ agonist rosiglitazone, but not by other agonists. These data demonstrate that the dual up-regulation of genes involved in both lipid catabolism and anabolism we observed is dependent on several PPAR isotypes in Syrian hamsters.

## Discussion

**FIGURE 5:**
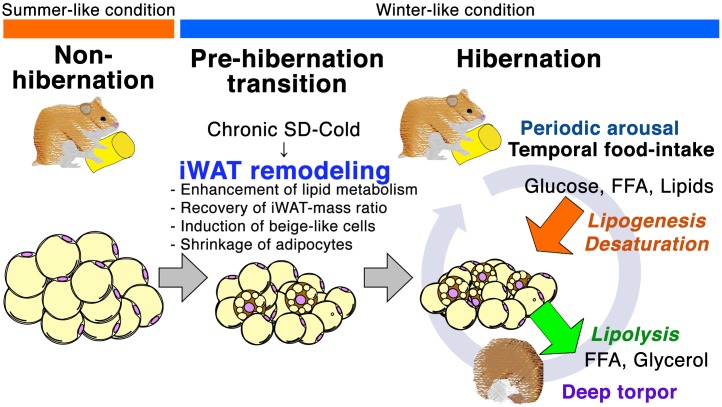
Schematic representation of iWAT remodeling for hibernation in Syrian hamster. Prolonged SD-Cold stimuli induce iWAT remodeling prior to initiating hibernation in Syrian hamsters. The morphology and cell population of adipocytes in iWAT changes during the transition from the non-hibernation to the hibernation state. Small adipocyte population increases, and beige-like cells emerge during Pre-HIB and HIB. Prolonged SD-Cold stimuli also alter the mRNA expression of genes in the insulin-PPAR signaling pathway. Up-regulation of lipid metabolism genes before the onset of hibernation could enhance the capacity for both lipid catabolism and anabolism in iWAT to prepare for metabolic challenges during the HIB. Thus, seasonal cues mediated by the ambient temperature and photoperiod alter the mTORC1-PPAR signaling network and induce iWAT remodeling prior to hibernation.

In the present study, we identified that transferal of female Syrian hamsters to winter-like conditions triggers pre-hibernation remodeling of iWAT at the cellular and molecular level (Figure 5). This initially manifests as a reduction in iWAT mass, although the iWAT-to-body mass ratio stabilizes and then increases over the following 2 months. This biphasic response of iWAT is likely explained by our finding that mRNA expression of lipogenic genes is induced with similar timing. The up-regulation of *Gpam*, a rate-limiting enzyme of triglyceride synthesis, may prevent over-shrinkage of white adipocytes and limit lipodystrophy by promoting lipogenesis before the onset of hibernation and during HIB. The simultaneous induction of lipid catabolism and anabolism genes in iWAT may be a phenotype that is unique to hamster hibernation (Baumber and Denyes, 1963); Syrian hamsters do not fatten during the pre-HIB under SD-Cold, and reduce their body mass below approximately 140 g (Chayama et al., 2016). They hoard food before the onset of hibernation (Larkin et al., 2002), and during PA states they do ingest small amounts of food. This food intake enables them to replenish glucose and FAs, which are then used to synthesize triglycerides. Thus, Syrian hamsters would require enhancement of lipogenesis to quickly synthesize and maintain sufficient lipids in WAT as a fuel during HIB. This is in striking contrast to the fat-storing species (ground squirrels, bears, marmots, dormice, etc.), which fatten by hyperphagia from late summer to fall and become anorexic in winter to survive without hoarding and eating food (Boswell et al., 1994; Dark, 2005; Melvin and Andrews, 2009).

Several possible mechanisms of simultaneous enhancement of lipid catabolism and anabolism pathway in iWAT can be considered. From Pre-HIB to HIB, a part of cells may activate lipid catabolism to supply fatty acids and generate heat, and the others may be anabolic to replenish lipid storage in iWAT. Previous reports showed that sympathetic nervous system and humor factors can temporarily activate the lipolysis and FAO in WAT (Braun et al., 2018; Caron et al., 2018). Such regulatory mechanism might switch lipid anabolic state to catabolic state in a spatiotemporally manner within iWAT, synchronizing to the torpor-arousal cycles during hibernation. Alternatively, lipid catabolism and anabolism pathways might be enhanced in the same cells. One possible explanation for simultaneous enhancement of lipogenesis and lipolysis comes from a recent study in a mouse model demonstrating that adipocytes are protected from lipid-induced endoplasmic reticulum (ER) stress by a re-esterification of fatty acids to triglyceride, which is mediated by diacylglycerol *O*-acyltransferase 1 (DGAT1) during lipolysis (Chitraju et al., 2017). In fact, we found that expression of *Dgat1* mRNA was significantly up-regulated during HIB (Figure 4A). Enhancement of re-esterification of fatty acids might also contribute to lowering oxidative stress levels, which is speculated to increase during hibernation, notably during PA because of the preferentially and highly retained polyunsaturated fatty acids (PUFA) during HIB (Arnold et al., 2011, 2015). Thus, hibernation accompanying active lipolysis may require protective mechanisms from lipotoxicity.

Several lines of evidence indicate that seasonal alteration in composition of lipids and FAs in the cellular membrane and stored fat is an important feature of mammalian hibernation. Increased dietary intake of unsaturated FAs can affect the length of torpor, metabolic rates, and minimum *Tb* tolerated by hibernators (reviewed in Arnold et al., 2015). It is proposed that high proportions of linoleic acid (C18:2 n-6) in phospholipids of sarcoplasmic reticulum (SR) in the heart improve cardiac function at low body temperature by enhancing the SR Ca^2+^ ATPase 2a (SERCA2a) activity during hibernation in Syrian hamsters and garden dormice (Arnold et al., 2011; Giroud et al., 2013, 2018). These studies also demonstrated diet-independent remodeling of tissue lipid composition, which can be in line with our finding that Syrian hamsters enhance *de novo* synthesis of unsaturated FAs and lipids in WAT by up-regulation of FA desaturases, *Scd1* and *Fads6* during Pre-HIB and HIB (Figure 3A). Such enhancement of *de novo* synthesis of unsaturated FAs from saturated FAs in iWAT might contribute to systemic alteration of FA composition in cellular membrane, and increased tolerance of cells and tissues under low body temperature during hibernation (Arnold et al., 2011). It could also help thermogenesis in BAT during hibernation, as BAT preferentially utilizes specific unsaturated FAs stored in WAT as an energy source for thermogenesis during rewarming from torpor (Carneheim et al., 1989; Jefimow and Wojciechowski, 2014). Furthermore, specific unsaturated FAs and lipids could work as lipokines, endogenous ligands for PPARγ in WAT (Mottillo et al., 2014). Further analysis will be needed for better understandings of the significance of enhanced *de novo* synthesis of unsaturated FAs in iWAT during Pre-HIB and HIB in Syrian hamsters.

The mTORC1-PPAR signaling network seems to be an important regulatory mechanism to adapt energy metabolism for hibernation (Buck et al., 2002; Eddy and Storey, 2003; El Kebbaj et al., 2009). Our data indicated that PPAR-γ agonist treatment simultaneously induced mRNAs of lipid catabolism and anabolism genes in iWAT explants of Syrian hamsters. The up-regulation of mTORC1 complexes during the period from pre-hibernation to hibernation that we observed may increase PPAR-γ levels, and consequently levels of its downstream target genes, and prevent excessive loss of fat mass (Polak et al., 2008; Laplante and Sabatini, 2009; Blanchard et al., 2012; Cai et al., 2016). However, it was reported that plasma insulin levels decrease under short photoperiodic conditions and during hibernation in Syrian hamsters (Weitten et al., 2013; Chakir et al., 2015). These lines of evidence imply that mTORC1 may be activated independently of insulin-PI3K-Akt signaling during Pre-HIB and HIB in iWAT of Syrian hamsters. Although future studies are required in order to elucidate the mechanisms responsible for the enhancement of mTORC1 activity, there are several possible candidates reported in the literature. Amino acids such as leucine can activate mTORC1 in an insulin-independent manner (Roh et al., 2003); leucine directly binds sestrin2 and suppresses its inhibitory effect on mTORC1 (Wolfson et al., 2016). Alternatively, serotonin may activate mTORC1 and PPARs in peripheral tissues of Syrian hamster during hibernation (Talaei et al., 2011; Talaei, 2014), because serotonin is reported to be an endogenous ligand for PPAR-γ (Waku et al., 2010) and the activation of serotonin receptor promotes lipogenesis in WAT of mice (Oh et al., 2015). Moreover, previous studies suggest that melatonin transduces photoperiodic stimulation to a neuroendocrine signal and could therefore control sexual activity, body mass, and hibernation in Syrian hamsters (Bartness and Wade, 1984; Vanecek et al., 1984; Pitrosky et al., 2003), which might be an upstream machinery of iWAT remodeling during the pre-HIB.

Another important finding in the present study is the emergence of beige-like cells in the iWAT of Syrian hamsters after prolonged exposure to SD-Cold stimuli (Figure 2). The beige-like cells in WAT may contribute to local thermogenesis (Okamatsu-Ogura et al., 2013; Bartesaghi et al., 2015). The enhancement of mTORC1 and PPAR signaling pathways may also recruit and activate beige-like cells in iWAT (Harms and Seale, 2013; Liu et al., 2016). Because lipolysis and FAO are required for cold-induced thermogenesis of brown and beige adipocytes in mice and humans (Lee et al., 2015; Blondin et al., 2017), the up-regulation of lipid catabolic genes in iWAT may reflect the emergence and activation of beige-like cells in the tissue during hibernation.

A recent study reported that beige cells can generate heat through UCP1-independent mechanism by activating SERCA2 pathway (Ikeda et al., 2017); SERCA2 pathway highly depends on glucose oxidation and activates Ca^2+^ cycling in endoplasmic reticulum for thermogenesis, although the UCP1 pathway mainly utilizes FAs and dissipate chemical energy in mitochondria as heat. Our data demonstrated that the *Atp2a2* mRNA (SERCA2) significantly increased after prolonged SD-Cold and during hibernation, while the *Ucp1* mRNA gradually decreased toward the onset of hibernation after prolonged SD-Cold in iWAT (Figure 2). The enhancement of SERCA2 pathway could be an effective strategy for simultaneously activating thermogenesis and FA synthesis in iWAT during PA; the FAs and triglycerides synthesized during PA may be saved in iWAT for the next torpor, by rewiring energy metabolism from FAO toward glucose oxidation for thermogenesis through SERCA2 pathway in beige-like cells during PA.

In Syrian hamsters, only a small proportion of cells were recruited to beige-like cells within iWAT, as is evident from our histological analysis compared to that of mice under similar environmental conditions (de Jong et al., 2015). One possible explanation is that Syrian hamsters preferentially retain white adipocytes to store a fuel for hibernation, rather than converting them into beige-like cells which may consume energy for heat-production. Indeed, hamsters establish large amount of BAT for hibernation (Kitao and Hashimoto, 2012), and may not require extra thermogenic cells in iWAT.

Our data also demonstrates Syrian hamsters provide a suitable model for monitoring the molecular physiology associated with pre-hibernation remodeling during the transition from a summer-like, non-hibernation state to a winter-like, hibernation state. Tracing the timing of the onset of body remodeling in these animals can be achieved simply by controlling the environmental condition (photoperiod and ambient temperature) (Jansky et al., 1984; Chayama et al., 2016), independent of circannual rhythms that define the timing of body remodeling and hibernation in some strictly seasonal hibernators (Kondo, 1987; Grabek et al., 2011; Olson et al., 2013; Hindle and Martin, 2014). We anticipate that this model will help elucidate the molecular pathways involved in pre-hibernation remodeling of lipid metabolism and whole body homeostasis. In addition to providing new insight into the amazing powers of hibernators, such information will also further our understanding of the metabolism and physiology of non-hibernators, including humans.

## Author Contributions

YC and YY conceived the experiments. YC, LA, DA, YS, TF, HT, and YT performed the experiments. YC, LA, YS, and YY analyzed data and wrote the manuscript. SS supervised RNA-seq analysis. MM and YY supervised the study.

## Conflict of Interest Statement

The authors declare that the research was conducted in the absence of any commercial or financial relationships that could be construed as a potential conflict of interest.

## Footnotes

1 https://www.ncbi.nlm.nih.gov/tools/primer-blast/

2 https://www.ncbi.nlm.nih.gov/assembly/GCF_000349665.1/

3 https://software.broadinstitute.org/morpheus

## Abbreviations

BAT: brown adipose tissue
HIB: hibernation period
HIB-DT: deep torpor during HIB
HIB-PA: periodic arousal during HIB
iWAT: subcutaneous inguinal WAT
LD: Long day
Non-HIB: non-hibernation period
PA: periodic arousal
Post-HIB: post-hibernation period
Pre-HIB: pre-hibernation period
SD: short day
WAT: white adipose tissue
