# Supplementary material for: Molecular Basis of White Adipose Tissue Remodeling That Precedes and Coincides With Hibernation in the Syrian Hamster, a Food-Storing Hibernator
Source: Front Physiol. 2019 Jan 28;9:1973. doi: 10.3389/fphys.2018.01973 (PMC6360343; doi:10.3389/fphys.2018.01973)
Supplement: Supplementary file 1 [file Data_Sheet_1.pdf]

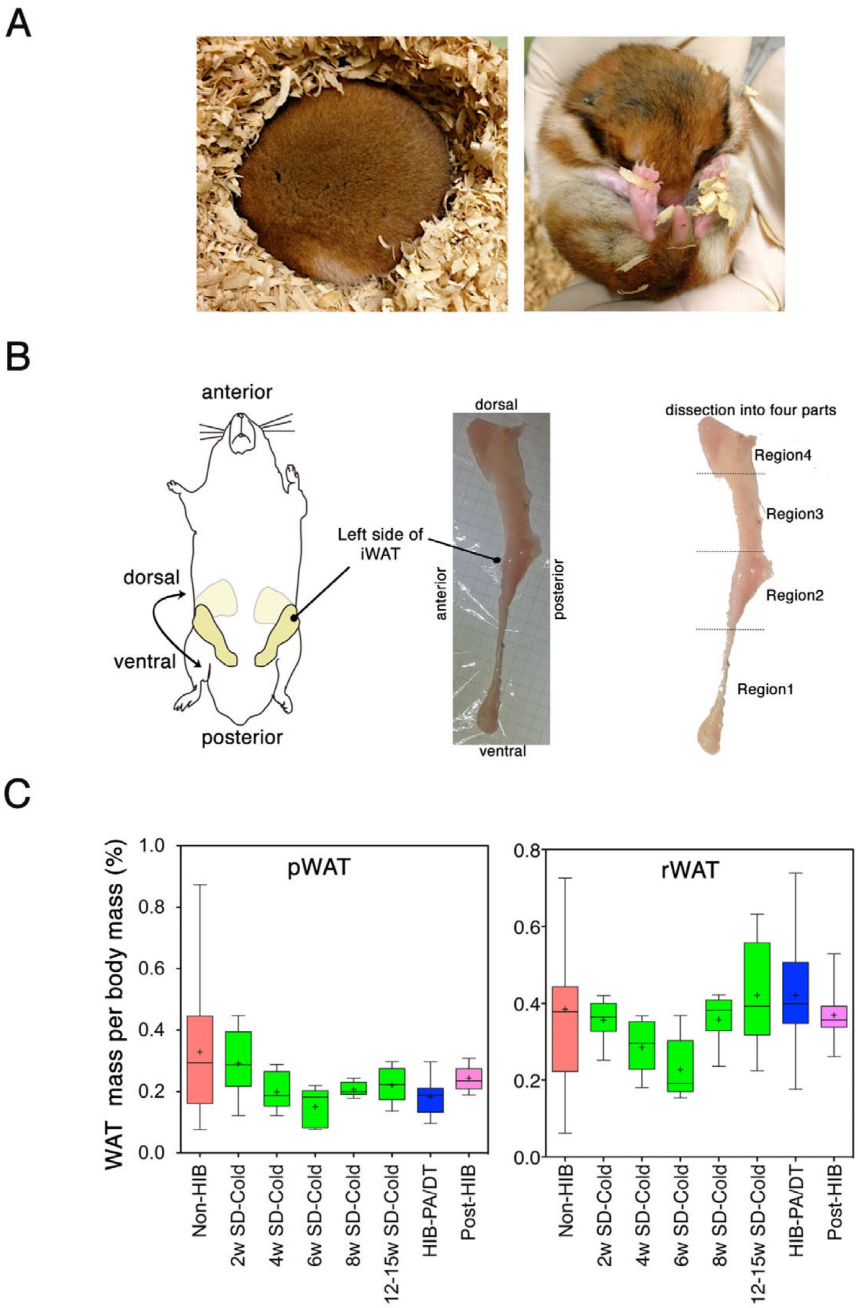


**Supplementary Figure S1**

(A) Dorsal view (left) and ventral view (right) of a Syrian hamster in deep torpor during HIB. (B) Anatomical location and morphology of iWAT in Syrian hamsters. Isolated iWAT was divided into four regions for histological analysis. (C) Mass of perigonadal WAT (pWAT) and retroperitoneal WAT (rWAT) before and after SD-Cold exposure. They tended to be gradually decreased during 6 weeks after the exposure and rWAT seemed to be recovered before hibernation.

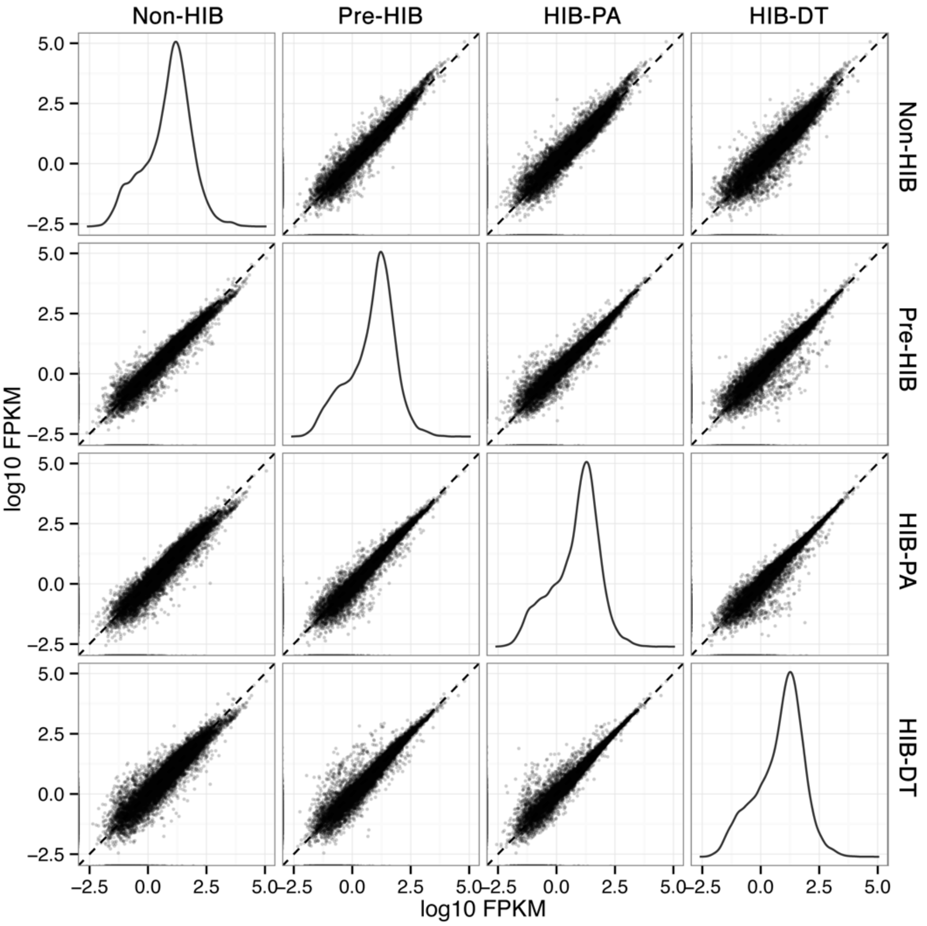


**Supplementary Figure S2**

Correlation plot of log10 FPKM values for genes between each combination of experimental groups. Each dot depicts a transcript. Although the majority of transcripts distributed on the lines representing similar expression levels between each state, a subset of gene transcripts deviated from the lines, which was differentially expressed in a state against its comparison.

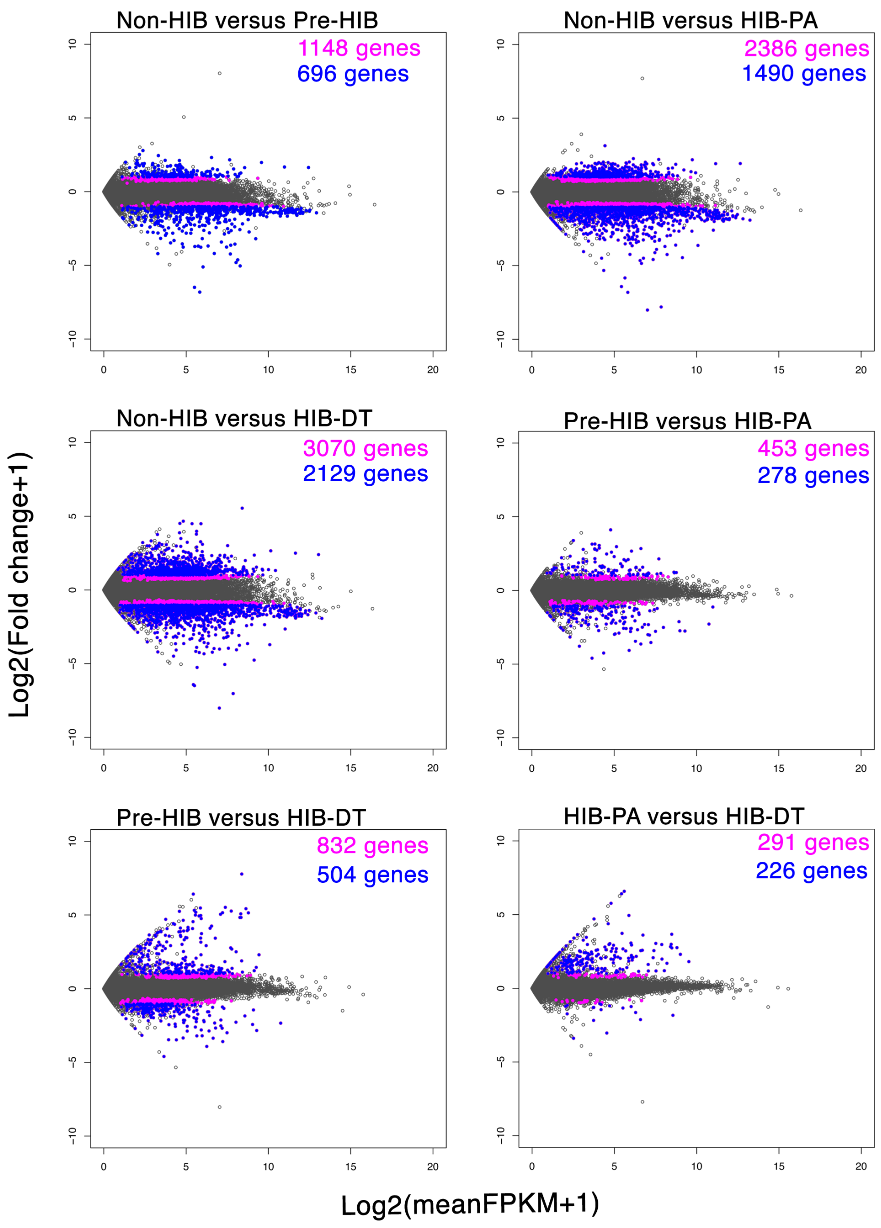


**Supplementary Figure S3**

MA plot of the log2 fold change of all genes. Magenta indicate differentially expressed genes (DEGs) with $q$-value less than 0.01. Blue indicate DEGs which exhibited more than twofold change between any groups with $q$-value $\leq$ 0.01.

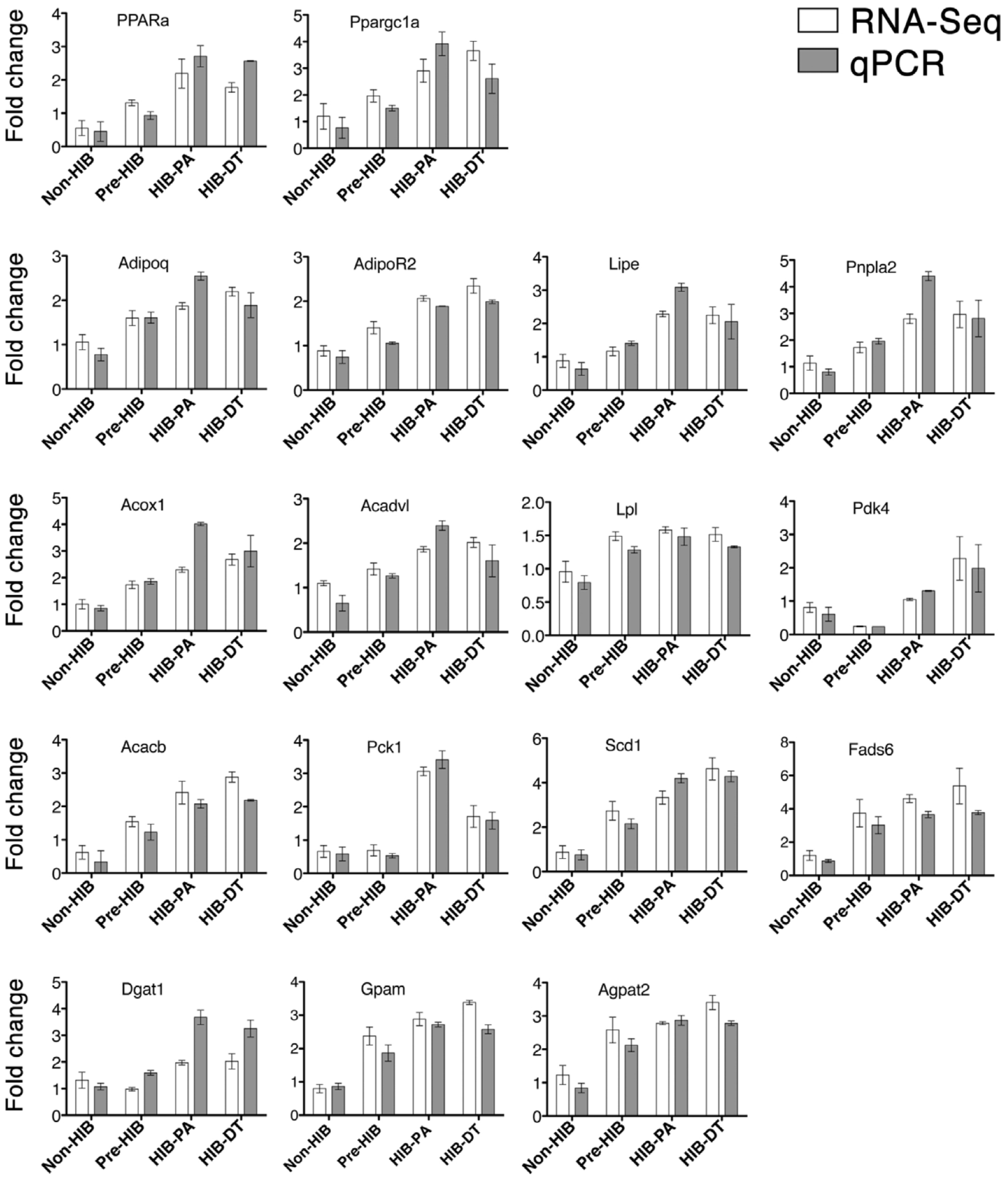


**Supplementary Figure S4**

qPCR validation of RNA-seq results for genes related to PPAR signaling, adipocytokine signaling, an lipid metabolism. RNA-seq fold change is shown in grey, and qPCR fold change is shown in white. Bar plots represent mean values of three animals for each group. Error bars represent the standard errors.

| experimental_group | # of reads | reads% |
| --- | --- | --- |
| Non-HIB_0 | 10.5M | 7.4 |
| Non-HIB_1 | 9.1M | 6.4 |
| Non-HIB_2 | 11.2M | 8 |
| Pre-HIB_0 | 12.9M | 9.1 |
| Pre-HIB_1 | 10.2M | 7.2 |
| Pre-HIB_2 | 8.4M | 5.9 |
| HIB-PA_0 | 11.0M | 7.8 |
| HIB-PA_1 | 14.5M | 10.3 |
| HIB-PA_2 | 13.0M | 9.2 |
| HIB-DT_0 | 13.1M | 9.3 |
| HIB-DT_1 | 9.9M | 7 |
| HIB-DT_2 | 17.5M | 12.4 |
| total | 141.3M | 100 |

**Supplementary Table S1**

Number and percentage of reads of each experimental replicate are shown.

| experimental_ group | Left reads input | Left reads mapped | Left read mapping rate | Right reads input | Right reads mapped | Right read mapping rate | Overall read mapping rate | Aligned pairs | concordant pair alignment rate |
|---|---|---|---|---|---|---|---|---|---|
| Non-HIB_0 | 10497844 | 9361593 | 89.2% | 10497844 | 9215922 | 87.8% | 88.5% | 8806077 | 82.5% |
| Non-HIB_1 | 9052271 | 8085261 | 89.3% | 9052271 | 7976527 | 88.1% | 88.7% | 7637573 | 82.9% |
| Non-HIB_2 | 11241435 | 9849686 | 87.6% | 11241435 | 9720276 | 86.5% | 87.0% | 9294496 | 81.1% |
| Pre-HIB_0 | 12909112 | 11462898 | 88.8% | 12909112 | 11279833 | 87.4% | 88.1% | 10770300 | 81.9% |
| Pre-HIB_1 | 10195308 | 9125317 | 89.5% | 10195308 | 8995870 | 88.2% | 88.9% | 8609289 | 83.2% |
| Pre-HIB_2 | 8373014 | 7477147 | 89.3% | 8373014 | 7349723 | 87.8% | 88.5% | 7022702 | 82.6% |
| HIB-PA_0 | 11035715 | 9790922 | 88.7% | 11035715 | 9638372 | 87.3% | 88.0% | 9234726 | 82.4% |
| HIB-PA_1 | 14546869 | 12993700 | 89.3% | 14546869 | 12809297 | 88.1% | 88.7% | 12248053 | 83.0% |
| HIB-PA_2 | 12957685 | 10167477 | 78.5% | 12957685 | 10017146 | 77.3% | 77.9% | 9517384 | 72.2% |
| HIB-DT_0 | 13081729 | 11640439 | 89.0% | 13081729 | 11443238 | 87.5% | 88.2% | 10936298 | 82.2% |
| HIB-DT_1 | 9907019 | 8823817 | 89.1% | 9907019 | 8695970 | 87.8% | 88.4% | 8282862 | 82.3% |
| HIB-DT_2 | 17535458 | 15264335 | 87.0% | 17535458 | 15024224 | 85.7% | 86.4% | 14329131 | 80.3% |

**Supplementary Table S2**

Mapping rate of left, right, and overall reads of each experimental replicate are shown.

A

| Term | Count | % | P-Value | Genes |
|---|---|---|---|---|
| hsa04910:Insulin signaling pathway | 7 | 0.262 | 0.02471 | PRKAR2A, EXOC7, RHOQ, MTOR, RPS6, RAPGEF1, CALM2 |
| hsa04640:Hematopoietic cell lineage | 5 | 0.187 | 0.054938 | CD38, CD3G, CD3D, CD2, CD7 |

B

| Term | Count | % | P-Value | Genes |
|---|---|---|---|---|
| hsa04662:B cell receptor signaling pathway | 11 | 0.252 | 2.27E-06 | PIK3CG, RAC2, RASGRP3, NFKBIE, PIK3CD, PLCG2, NFAT5, CD22, PIK3AP1, BLNK, SYK |
| hsa04660:T cell receptor signaling pathway | 9 | 0.206 | 0.001518 | PIK3CG, ITK, CD8A, NFKBIE, PIK3CD, CBL, NFAT5, MAP3K14, LCP2 |
| hsa04670:Leukocyte transendothelial migration | 9 | 0.206 | 0.002666 | PIK3CG, ITK, RAC2, PIK3CD, PLCG2, MYLPF, TXK, ACTN3, MYL9 |
| hsa04666:Fc gamma R-mediated phagocytosis | 7 | 0.16 | 0.01267 | PIK3CG, DOCK2, RAC2, PIK3CD, PLCG2, PPAP2A, SYK |
| hsa04062:Chemokine signaling pathway | 10 | 0.229 | 0.013731 | PIK3CG, ITK, DOCK2, CCR6, RAC2, CXCR5, PREX1, PIK3CD, CXCR2, PLCB2 |
| hsa04370:VEGF signaling pathway | 6 | 0.137 | 0.018093 | PIK3CG, RAC2, PIK3CD, PLCG2, NFAT5, HSPB1 |
| hsa04664:Fc epsilon RI signaling pathway | 6 | 0.137 | 0.021097 | PIK3CG, RAC2, PIK3CD, PLCG2, LCP2, SYK |
| hsa00250:Alanine, aspartate and glutamate metabolism | 4 | 0.092 | 0.02467 | ADSSL1, GFPT1, IL4I1, GPT |

**Supplementary Table S3**

(A and B) KEGG pathway analysis results for the top 300 genes that contributed most to PC1 (A) and PC2 (B).

| Term | Count | % | PValue | Genes |
|---|---|---|---|---|
| hsa03010:Ribosome | 56 | 5.19 | 2.13E-40 | RPL18, RPL17, RPL36A, RPL19, RPS27L, RPL22L1, RPS3, RPS3A, RPLP1, RPL10, FAU, RPL12, RPS27A, RPS4X, RPS18, RPS19, RPS16, RPS15, RPS12, RPS13, RPS10, RPS11, UBA52, RPL27A, RPS15A, RPL36, RPL37, RPL38, RPS25, RPS26, RPL30, RPS27, RPS28, RPL32, RPL7, RPL9, RPL34, RPL3, RPL5, RPL10A, RPL4, RPS23, RPSA, RPL26, RPL27, RPL23A, RPL24, RPS6, RPS8, RPL28, RPL29, RPS7, RPL23, RPL13A, RPL22, RPL21 |
| hsa05340:Primary immunodeficiency | 13 | 1.205 | 6.94E-06 | CIITA, PTPRC, CD8A, CD3D, CD3E, CD40LG, TNFRSF13C, IL2RG, CD40, IL7R, ADA, BLNK, BTK |
| hsa04620:Toll-like receptor signaling pathway | 18 | 1.668 | 0.001861695 | PIK3CG, LY96, TBK1, TLR1, PIK3CD, TLR2, TLR3, TLR4, CD40, TLR6, TLR7, CXCL10, FOS, CD86, CD80, TICAM2, IL1B, SPP1 |
| hsa04060:Cytokine-cytokine receptor interaction | 35 | 3.244 | 0.00197654 | IL1R2, CCL2, CCR1, IL18, IL21R, CCL8, CXCR3, IL7R, TNFRSF4, CXCL10, CCL24, CXCR5, CXCR4, TNFRSF18, IL1B, IL2RG, FAS, IL13RA1, LTB, CD27, FLT3, TNFRSF13C, CCL19, HGF, CD40, CCL11, TNFRSF9, CCR7, CCR6, CCR5, RELT, CXCL14, CXCL13, CD40LG, CCR2 |
| hsa04640:Hematopoietic cell lineage | 16 | 1.483 | 0.002370268 | IL1R2, CD3G, CD3D, CD8A, FLT3, CD3E, FCER2, MME, ITGA4, IL7R, CD38, MS4A1, CD2, CD22, IL1B, CD7 |
| hsa04062:Chemokine signaling pathway | 26 | 2.41 | 0.005127849 | CCL2, FGR, CCR1, CCL8, GNG11, CXCR3, CXCL10, CCL24, CXCR5, CXCR4, GNG2, SHC2, PIK3CG, ITK, HCK, PIK3CD, CCL19, CCL11, CCR7, CCR6, CCR5, CXCL14, CXCL13, GNG10, CCR2, RAP1A |
| hsa04110:Cell cycle | 18 | 1.668 | 0.016467932 | CDK1, CDC14A, RBL1, TTK, ATR, PTTG1, CDC26, MCM4, ATM, RBX1, CCNB1, CCNB2, MAD2L1, PCNA, BUB1, BUB1B, CCNA2, STAG2 |
| hsa00520:Amino sugar and nucleotide sugar metabolism | 9 | 0.834 | 0.018832504 | GALK2, PGM3, UAP1, GNPDA1, GNPDA2, HK3, HEXB, NPL, NANP |
| hsa05310:Asthma | 7 | 0.649 | 0.022176973 | FCER1A, CCL11, CD40LG, PRG2, FCER1G, MS4A2, CD40 |
| hsa04662:B cell receptor signaling pathway | 12 | 1.112 | 0.029791741 | PIK3CG, FOS, DAPP1, RASGRP3, NFKBIE, PIK3CD, CD22, PIK3AP1, CD79B, CD72, BLNK, BTK |
| hsa00740:Riboflavin metabolism | 5 | 0.463 | 0.031351503 | TYR, ENPP3, ACP5, MTMR7, PHPT1 |
| hsa04660:T cell receptor signaling pathway | 15 | 1.39 | 0.040135789 | PIK3CG, ITK, PTPRC, CD3G, CD3D, CD8A, NFKBIE, CD3E, PIK3CD, CD247, FOS, LAT, CD40LG, NCK1, GRAP2 |

**Supplementary Table S4**

KEGG pathway analysis results for Cluster 8 (1528 genes) in Figure 3D.

| Term | Count | % | PValue | Genes |
|---|---|---|---|---|
| hsa04510:Focal adhesion | 34 | 3.895 | 2.34E-08 | TLN1, TLN2, DIAPH1, BCAR1, ITGB4, MYL9, LAMB2, DOCK1, LAMB1, THBS1, RAPGEF1, THBS2, COL4A2, TNXB, FLT1, COL4A1, ACTN4, ITGA3, FLNB, FLNA, KDR, LAMA2, VWF, LAMA1, LAMA3, ITGA6, LAMA5, JUN, ITGA7, PDGFRB, MAPK9, RELN, LAMC1, PARVA |
| hsa04512:ECM-receptor interaction | 20 | 2.291 | 1.63E-07 | COL4A2, TNXB, COL4A1, DAG1, ITGB4, HSPG2, ITGA3, LAMA2, LAMA1, VWF, LAMA3, LAMB2, ITGA6, LAMA5, ITGA7, RELN, LAMC1, LAMB1, THBS1, THBS2 |
| hsa03320:PPAR signaling pathway | 14 | 1.604 | 1.19E-04 | CPT1B, PPARA, ACOX1, SLC27A1, RXRA, SCD, EHHADH, CPT1A, PCK1, ACOX3, SORBS1, HMGCS2, PLIN1, ANGPTL4 |
| hsa04270:Vascular smooth muscle contraction | 18 | 2.062 | 1.87E-04 | ADCY3, ADCY4, ACTA2, GNA11, ADCY5, ADCY6, GNA12, PRKCE, ARHGEF12, ITPR2, ARHGEF11, MYL9, GNAQ, ADCY9, MYH11, PLA2G6, ADRA1A, CACNA1D |
| hsa05200:Pathways in cancer | 36 | 4.124 | 2.14E-04 | FGFR1, FOXO1, ZBTB16, TCF7L2, TCF7L1, LAMB2, CDKN2B, LAMB1, FGF1, TRAF3, APC, COL4A2, PLD1, BCR, COL4A1, EPAS1, RXRA, FZD1, ITGA3, FZD4, FZD7, LAMA2, JUP, CCDC6, LAMA1, CDKN1A, LAMA3, ITGA6, LAMA5, JUN, PDGFRB, MAPK9, PTCH1, MTOR, LAMC1, ABL1 |
| hsa05222:Small cell lung cancer | 14 | 1.604 | 8.82E-04 | COL4A2, COL4A1, RXRA, ITGA3, LAMA2, LAMA1, LAMA3, LAMB2, ITGA6, CDKN2B, LAMA5, LAMC1, LAMB1, TRAF3 |
| hsa04520:Adherens junction | 13 | 1.489 | 0.001299535 | PTPRB, FGFR1, PTPRM, ACTN4, SORBS1, BAIAP2, WASF1, WASF2, LMO7, TCF7L2, MLLT4, TCF7L1, FARP2 |
| hsa04920:Adipocytokine signaling pathway | 12 | 1.375 | 0.001326729 | CPT1B, PPARA, IRS2, RXRA, ADIPOR2, MAPK9, MTOR, ACACB, PPARGC1A, CPT1A, PCK1, PTPN11 |
| hsa04910:Insulin signaling pathway | 18 | 2.062 | 0.001685581 | IRS2, HK2, FOXO1, RHOQ, IGF2, ACACB, PPARGC1A, RPTOR, PCK1, PRKAR2A, SORBS1, GYS1, FASN, MAPK9, SH2B2, MTOR, RAPGEF1, LIPE |
| hsa04810:Regulation of actin cytoskeleton | 24 | 2.749 | 0.002597504 | FGFR1, SSH1, ACTN4, DIAPH1, BAIAP2, BCAR1, WASF1, WASF2, GNA12, ITGB4, IGF2, ITGA3, ARHGEF12, MYL9, DOCK1, ITGA6, ITGA7, PDGFRB, MYH14, FGF1, PIP4K2C, MYH10, SLC9A1, APC |
| hsa04912:GnRH signaling pathway | 14 | 1.604 | 0.003674139 | ADCY3, ADCY4, PLD1, GNA11, ADCY5, ADCY6, ITPR2, MAP3K4, GNAQ, ADCY9, JUN, PLA2G6, MAPK9, CACNA1D |
| hsa05412:Arrhythmogenic right ventricular cardiomyopathy (ARVC) | 12 | 1.375 | 0.00373146 | JUP, LAMA2, ATP2A2, ACTN4, ITGA6, ITGA7, DAG1, ITGB4, ITGA3, CACNA1D, TCF7L2, TCF7L1 |
| hsa00564:Glycerophospholipid metabolism | 11 | 1.26 | 0.004988476 | GPD2, CDS2, CPT1B, GPD1, PLD1, LCAT, PLA2G6, PHOSPHO1, GPAM, PNPLA3, AGPAT2 |
| hsa05414:Dilated cardiomyopathy | 13 | 1.489 | 0.005938652 | ADCY3, LAMA2, ADCY4, ITGA6, ADCY9, ATP2A2, ADCY5, ADCY6, ITGA7, DAG1, ITGB4, ITGA3, CACNA1D |
| hsa04360:Axon guidance | 16 | 1.833 | 0.006636561 | ABLIM1, PLXNA2, NTN4, L1CAM, DPYSL2, ARHGEF12, NTN1, EPHB1, SEMA6A, EPHA4, SEMA6B, UNC5B, SEMA3G, SRGAP3, EFNA5, ABL1 |
| hsa04144:Endocytosis | 20 | 2.291 | 0.008728296 | PLD1, FLT1, LDLR, ASAP2, ASAP1, CXCR2, ASAP3, KDR, HSPA1L, RAB11FIP3, HSPA2, ARRB1, PDCD6IP, SMURF1, GRK5, AGAP1, ARAP3, EHD2, IQSEC1, EPN2 |
| hsa02010:ABC transporters | 8 | 0.916 | 0.011732413 | ABCB9, ABCC3, ABCC4, ABCB10, ABCB1, ABCG4, ABCB6, ABCA3 |
| hsa04916:Melanogenesis | 12 | 1.375 | 0.025562408 | ADCY3, ADCY4, GNAO1, GNAQ, ADCY9, ADCY5, ADCY6, FZD1, FZD4, TCF7L2, TCF7L1, FZD7 |
| hsa04710:Circadian rhythm | 4 | 0.458 | 0.034885909 | CRY2, PER2, PER1, PER3 |

## Supplementary Table S5

KEGG pathway analysis results for Cluster 5 (990 genes) in Figure 3D.

| Gene | Forward (5'->3') | Reverse (5'->3') | NCBI Reference Sequence |
|---|---|---|---|
| PPARa | AAGCTGTCCAGGCTCCGAGG | CGGGGCAGCTGACTGATGAGG | NM_001281543.1 |
| Ppargc1a | AAGGGGCTGGTTGCCTGCAT | CATCCAGCTCCTGAATGACGCC | XM_005068779.3 |
| Adipoq | AGGAGAGCCGGGAGAAAGCG | AACGCTGGGGACGGTGACTC | XM_021227970.1 |
| AdipoR2 | GGCAGATCGGCTGGCTAATGC | ACGTTCTGGGATGCGGGCAG | XM_021224762.1 |
| Lipe | GCATTGTGCCCTGCTCGGTT | CAGTGACGCAGAGGTTGCCG | XM_013125988.2 |
| Pnpla2 | GCAGGGCTACAGAGACGGACT | TTTCCTGGGGGACAACCGGG | XM_005064203.3 |
| Acox1 | CGAGGGCGACCTGAGCCTTT | GCTCTTGCTGCTCTGCGGTG | XM_005069904.2 |
| Acadvl | GCAGGCGGGACGTAAGAGGA | CCGCCCCACACTCGGAGTTA | XM_005067510.3 |
| Lpl | TCCATGGCTGGACGGTGACA | CGATGACGTTGGAGTCGGGC | XM_013117159.2 |
| Pdk4 | CGGCAAGAGTTGCCCGTGAG | CAGCTGCACGGAGGGGGTAT | XM_005082915.3 |
| Acacb | GGACAGCTGAAGGCCCGAGA | CCAGCATGTGGCCTGGTGTG | XM_021232318.1 |
| Pck1 | CCAAAACCGGCCTCAGCCAG | TTCATGCACCCCGGGAACCT | XM_005074472.3 |
| Scd1 | ACGGGAGAAGCAGAAGACCGT | CCTGGTAGCTGGGGTCGTGT | NM_001281585.1 |
| Fads6 | GAAGGTGCCGTGCCTCAACC | AACGCTCGAGAGCGACCAGT | XM_005069942.3 |
| Dgat1 | GCCGAAGAGGAGGTGCGAGA | CTTTGTCCGGGGTCGGGACT | XM_005085991.3 |
| Gpam | CTCGACGAGACCCCAGACGG | GGAACTGCTGCTGCCTGAGC | XM_013111923.2 |
| Agpat2 | GGAGCTGGGGGAAGACAGGT | GTGGGGAGCAGGATAAGGCGT | XM_005083644.3 |
| Ucp1 | GGGCAACCTACCGAGGTCGT | GGTCCCAGTGTAGCGGGGTT | NM_001281332.1 |
| Prdm16 | CATGGGCTTCGACCACACCC | GCCCCTTCCCAAAGGTTGGC | XM_021232554.1 |
| Atp2a2 | GCTCACACAAAGACGGTGGAG | CCCATCTCTCCTTGAGCTTCT | ENSMAUT00000028608.1 |
| Ppia1 | GCAGACAAAGTTCCAAAGACAGCA | TCCTGGGATAATTCTGTGAAAGGAG | XM_005086775.3 |

**Supplementary Table S6**

List of real-time PCR primers listed 5’ to 3’.

| Gene Symbol | Gene Name |
|---|---|
| 4E-BP2 | Eukaryotic translation initiation factor 4E binding protein 2 |
| Acacb | Acetyl-CoA carboxylase beta |
| Acot2 | Acyl-CoA thioesterase 2 |
| Acox1 | Acyl-CoA oxidase 1 |
| Adipoq | Adiponectin |
| AdipoR2 | Adiponectin receptor 2 |
| Agpat2 | 1-acylglycerol-3-phosphate O-acyltransferase 2 |
| Akt | v-akt murine thymoma viral oncogene homolog 1 |
| Angptl4 | Angiopoietin like 4. |
| Atp2a2 | ATPase sarcoplasmic/endoplasmic reticulum Ca2+ transporting 2 |
| Camkk2 | Calcium/calmodulin dependent protein kinase kinase 2 |
| Cd40 | CD40 molecule |
| Clstn2 | Calsyntenin 2 |
| Cpt1a/1b | Carnitine palmitoyltransferase 1 alpha/beta |
| Deptor | DEP domain containing MTOR interacting protein |
| Dgat1 | Diacylglycerol O-acyltransferase 1 |
| Ebf3 | EBF transcription factor 3. |
| Ehhadh | Enoyl-CoA hydratase and 3-hydroxyacyl CoA dehydrogenase |
| Eif4e3 | Eukaryotic translation initiation factor 4E family member 3. |
| Fads6 | Fatty acid desaturase 6 |
| Fasn | Fatty acid synthase |
| Fbxo31 | F-box protein 31 |
| Gpam | Glycerol-3-phosphate acyltransferase |
| Gpd1 | Glycerol-3-phosphate dehydrogenase 1 |
| Gys1 | Glycogen synthase 1 |
| Hmgcs2 | 3-hydroxy-3-methylglutaryl-CoA synthase 2 |
| Hspb7 | Heat shock protein family B (small) member 7 |
| InsR | Insulin receptor |
| Irs2 | Insulin receptor substrate 2 |
| Lamtor2/3/5 | Late endosomal/lysosomal adaptor 2/3/5，MAPK and MTOR activator 2/3/5 |
| Lipe | Lipase E hormone sensitive type |
| Mgll | Monoglyceride lipase |
| mTOR | Mechanistic target of rapamycin |
| Oplah | 5-oxoprolinase |
| Orm1 | Orosomucoid 1 |
| Pck1 | Phosphoenolpyruvate carboxykinase 1 |
| Pdk1 | Pyruvate dehydrogenase kinase 1 |
| Pdk4 | Pyruvate dehydrogenase kinase 4 |
| PI3K | Phosphoinositide 3-kinase |
| Plin1/5 | Perilipin 1/5 hormone sensitive type |
| Pnpla2/3 | Patatin like phospholipase domain containing 2/3 |
| Ppara | Peroxisome proliferator activated receptor alpha |
| Ppargc1a | Peroxisome proliferator-activated receptor gamma coactivator 1-alpha |
| Ppargc1b | Peroxisome proliferator-activated receptor gamma coactivator 1-beta |
| Prdm16 | PR domain 16 |
| Prkar2a | Protein kinase cAMP-dependent type II regulatory subunit alpha |
| Pten | Phosphatase and Tensin Homolog Deleted from Chromosome 10 |
| Pygl | Glycogen phosphorylase L |
| Rapgef1 | Rap guanine nucleotide exchange factor 1 |
| Raptor | Regulatory associated protein of MTOR complex 1 |
| Rheb | Ras homolog enriched in brain |
| Rhoq | Ras homolog family member Q |
| Rictor | RPTOR independent companion of MTOR complex 2 |
| Rragd | Ras related GTP binding D |
| Rxra | Retinoid X receptor alpha |
| Scd1 | Stearoyl-Coenzyme A desaturase 1 |
| Sesn2 | Sestrin 2 |
| Sh2b2 | SH2B adaptor protein 2 |
| Slc27a1 | Solute carrier family 27 member 1 |
| Sorbs1 | Sorbin and SH3 domain containing 1 |
| Sp100 | SP100 nuclear antigen |
| Sparcl1 | SPARC like 1 |
| Syne2 | Spectrin repeat containing nuclear envelope protein 2 |
| Tlr4 | Toll like receptor 4 |
| Tnfrsf9 | TNF receptor superfamily member 9 |
| Tsc2 | TSC complex subunit 2 |
| Ucp1 | Uncoupling protein 1 |

## Supplementary Table S7

Gene symbols and gene names described in this study.
